# SARS-CoV-2 vaccination induces mucosal antibody responses in previously infected individuals

**DOI:** 10.1038/s41467-022-32389-8

**Published:** 2022-09-01

**Authors:** Kaori Sano, Disha Bhavsar, Gagandeep Singh, Daniel Floda, Komal Srivastava, Charles Gleason, PARIS Study Group, Angela A. Amoako, Dalles Andre, Katherine F. Beach, Maria C. Bermúdez-González, Gianna Cai, Christian Cognigni, Hisaaki Kawabata, Giulio Kleiner, Neko Lyttle, Wanni Mendez, Lubbertus C. F. Mulder, Annika Oostenink, Ariel Raskin, Aria Rooker, Kayla T. Russo, Ashley Beathrese T. Salimbangon, Miti Saksena, Levy A. Sominsky, Johnstone Tcheou, Ania Wajnberg, Juan Manuel Carreño, Viviana Simon, Florian Krammer

**Affiliations:** 1 Department of Microbiology, Icahn School of Medicine at Mount Sinai, New York, NY USA; 2 Department of Pathology, Molecular and Cell Based Medicine, Icahn School of Medicine at Mount Sinai, New York, NY USA; 3 Division of Infectious Diseases, Department of Medicine, Icahn School of Medicine at Mount Sinai, New York, NY USA; 4 The Global Health and Emerging Pathogen Institute, Icahn School of Medicine at Mount Sinai, New York, NY USA; 5 Department of Internal Medicine, Icahn School of Medicine at Mount Sinai, New York, NY USA; 6 Department of Geriatrics and Palliative Medicine, Icahn School of Medicine at Mount Sinai, New York, NY USA

**Keywords:** RNA vaccines, Viral infection, SARS-CoV-2, Adaptive immunity

## Abstract

Immune responses at the respiratory mucosal interface are critical to prevent respiratory infections but it is unclear to what extent antigen specific mucosal secretory IgA (SIgA) antibodies are induced by mRNA vaccination in humans. Here we analyze paired serum and saliva samples from patients with and without prior coronavirus disease 2019 (COVID-19) at multiple time points pre and post severe acute respiratory syndrome coronavirus 2 (SARS-CoV-2) mRNA vaccination. Our results suggest mucosal SIgA responses induced by mRNA vaccination are impacted by pre-existing immunity. Indeed, vaccination induced a minimal mucosal SIgA response in individuals without pre-exposure to SARS-CoV-2 while SIgA induction after vaccination was more efficient in patients with a history of COVID-19.

## Abstract (web summary)

Prior exposure to infectious agents can impact the vaccination induced immune response. Here the authors show prior SARS-CoV-2 infection results in more efficient induction of mucosal SARS-CoV-2 secretory IgA antibody following mRNA vaccination.

## Introduction

Severe acute respiratory syndrome coronavirus 2 (SARS-CoV-2), a novel betacoronavirus, emerged in December 2019 as the causative agent of coronavirus disease 2019 (COVID-19). Since then, this virus has caused a pandemic that has so far claimed the lives of more than 5.2 million people worldwide^1^. Since SARS-CoV-2 infects the epithelium of the respiratory tract and causes pneumonia^2^, secretory IgA (SIgA) antibodies, which are present in the upper and lower respiratory tract, may play an important role in the defense against infection. SIgA antibodies are IgA antibodies produced and transported from the mucosal stroma to the mucosal surface by utilizing the polymeric immunoglobulin receptor (pIgR) expressed on the basolateral side of epithelial cells^3^. Upon release of IgA antibodies to the mucosal surface, the extracellular segment of the pIgR, the secretory component (SC) is incorporated into the IgA molecules, which is a characteristic feature of SIgA antibodies. Another characteristic of SIgA antibodies is that these are mostly present in the form of multimers, such as dimers. These multimeric IgA antibodies display higher anti-viral activity than monomeric IgA antibodies^4^. SIgA antibodies are known to provide immediate immunity by eliminating respiratory pathogens before they pass through the mucosal barrier^5,6^. For example, during influenza virus infections, it has been reported that the main player in influenza immunity from previously infected individuals may be SIgA in the upper respiratory tract^7^. This adaptive immune response against the influenza virus is primarily induced in secondary lymphoid tissues such as the mucosa-associated lymphoid tissue (MALT), which is responsible for the adaptive immune response induced at the virus infection and propagation site, and thus cannot be induced by injectable vaccines^3^. In the case of SARS-CoV-2, it has been reported that IgA antibodies that bind to the virus are rapidly produced, even before IgG antibodies, and can be detected in the serum and saliva of COVID-19 patients up to around 40 days post onset of symptoms as well^8–11^. A recent study also reports the detection of IgA antibodies in nasal secretions in COVID-19 patients^12^. However, it is unclear whether the currently used vaccines, including mRNA-based vaccines, which are intramuscularly administered, induce SIgA antibody responses. This study aims, thus, to determine whether antigen-specific SIgA are induced in response to COVID-19 mRNA vaccination.

## Results

### Anti-SARS-CoV-2 spike SIgA antibodies are detected in human saliva following vaccination

We used longitudinal serum and saliva samples collected from 29 adult study participants over a period of 200–372 days. 18/29 of the study participants (62%) were infected with SARS-CoV-2 on average 295.5 days prior to the first vaccine dose and were seropositive prior to vaccination (seropositive group). All the 18 seropositive participants had mild COVID-19 at the beginning of the pandemic (from March to April 2020), a time when only ancestral wild-type SARS-CoV-2 was circulating in New York City. 11/29 study participants (38%) had no previous SARS-CoV-2 infection history and were seronegative for SARS-CoV-2 antibodies prior to vaccination (seronegative group). Participants received either the Moderna mRNA-1273 vaccine or the Pfizer-BioNTech BNT162b2 vaccine two times. There were no differences in the demographics of the participants in the seropositive and seronegative groups (Supplementary Table 1**)**. Demographics of seropositive and seronegative study participants and sample collection timepoints from each individual are summarized in Supplementary Tables 2 and 3, respectively. Samples were collected at multiple time points prior to and after vaccination. We measured anti-SARS-CoV-2 spike binding IgG titers in serum samples and anti-SARS-CoV-2 spike IgG, SIgA, and nucleoprotein (NP) SIgA titers in saliva by enzyme-linked immunosorbent assay (ELISA). One of the challenges in the measurement of antigen-specific SIgA titers in saliva samples is the fact that monomeric IgA and IgG antibodies from serum leak into saliva via crevicular fluid^3^. Therefore, a detection antibody that only recognizes human SC-bound IgA antibodies was used to ensure specific detection of SIgA induced at the mucosa. Another challenge in the measurement of antigen-specific SIgA titers in the saliva is that the IgA concentration within saliva is variable not only between individuals but within samples collected from the same individual, depending on various factors such as circadian rhythm, stress, and collection method of saliva samples^3^. We measured, therefore, the total IgA concentration within each saliva sample and normalized the SARS-CoV-2-specific SIgA titers based on the total IgA content within each saliva sample. While this methodology may induce biases due to the presence of small amounts of serum IgA which leaks into the saliva via the crevicular fluid, it has been shown that this monomeric version of IgA amounts to only about 10% of IgA in saliva and we, therefore, think the bias is negligible^30^.

**Fig. 1:**
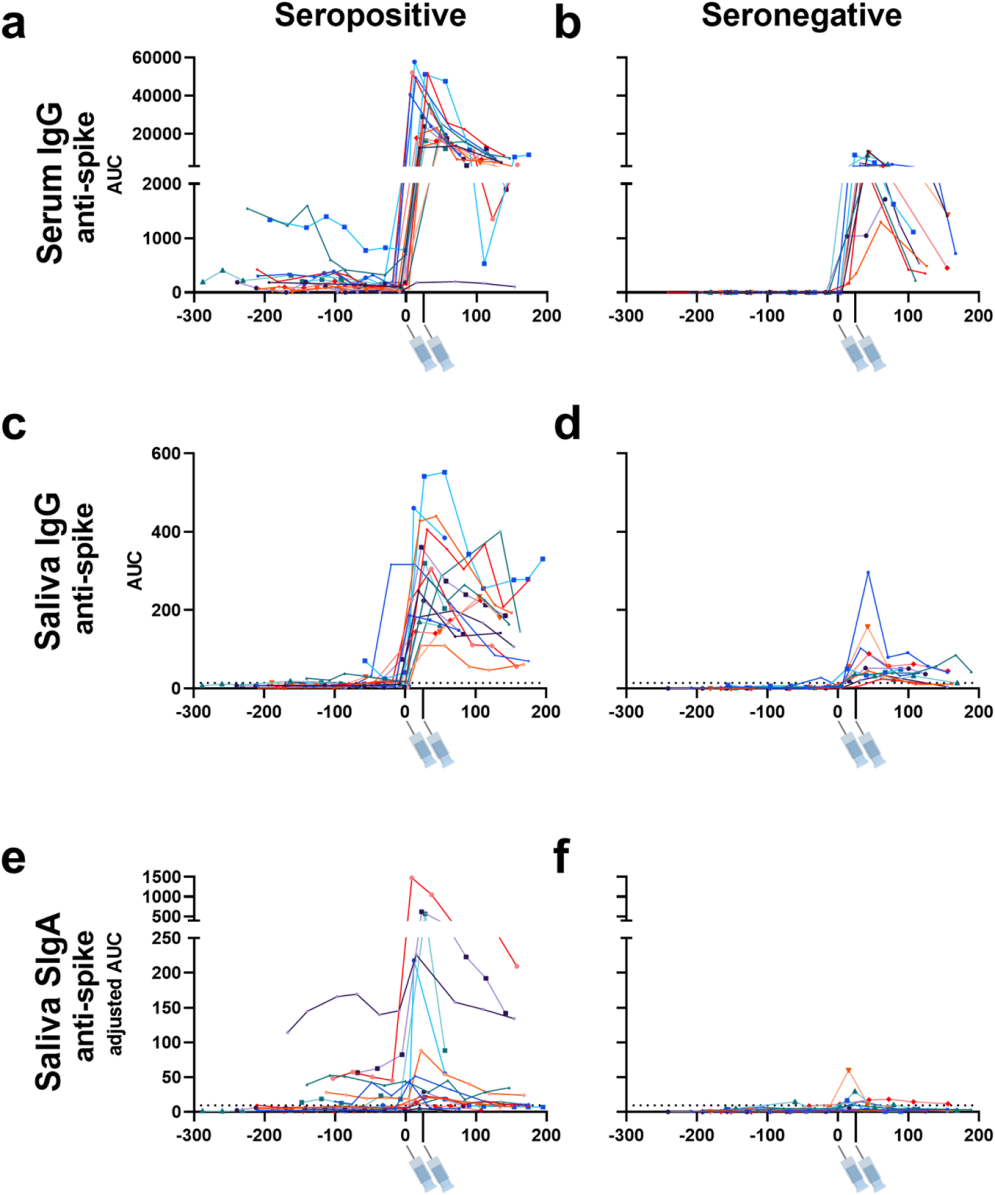
Time course of vaccine-induced serum and mucosal anti-SARS-CoV-2 antibody titers in adult participants with or without previous SARS-CoV-2 infection. Anti-SARS-CoV-2 spike IgG titers in serum (**a**, **b**), anti-SARS-CoV-2 spike IgG titers in saliva (**c**, **d**), and anti-SARS-CoV-2 spike SIgA titers in saliva (**e**, **f**) collected from 18 individuals with previous SARS-CoV-2 infection (seropositive) and 11 individuals without previous SARS-CoV-2 infection (seronegative) prior to and after vaccination. 6–13 different timepoints were assessed per individual. *X*-axis values represent days post-first vaccination and *Y*-axis values represent antibody titers calculated as area under the curve (AUC). Syringe symbols point to the approximate time when the first and second vaccine dose were administered (28 days for Moderna mRNA-1273 vaccine and 21 days for the Pfizer-BioNTech BNT162b2 vaccine). Dotted lines are cut-off values (mean+3 SD of the AUC of samples from seronegative individuals before vaccination). Different colors represent different individuals. This data is also presented on a logarithmic scale in Supplementary Fig. 1. Source data are provided as a Source Data file.

First, we evaluated the changes in anti-SARS-CoV-2 serum and saliva antibody titers before and after vaccination in each individual. An evident peak in anti-SARS-CoV-2 spike serum and mucosal antibody titers was observed in the seropositive group (Fig. 1a, c, e). In the seronegative group, peaks in serum and saliva anti-spike IgG titers could be observed, though these titers were low compared to the seropositive group (Fig. 1b, d). Interestingly, some individuals in the seronegative group presented a peak in anti-spike SIgA titers in saliva (Fig. 1f). The peak that was observed in anti-SARS-CoV-2 spike reflected the mucosal IgA response to vaccination, as most individuals did not demonstrate an increase in anti-SARS-CoV-2 NP SIgA which would indicate potential concurrent infection (Supplementary Fig. 2).

### Higher levels of anti-SARS-CoV-2 spike SIgA antibodies are induced in humans with prior virus infection

**Fig. 2:**
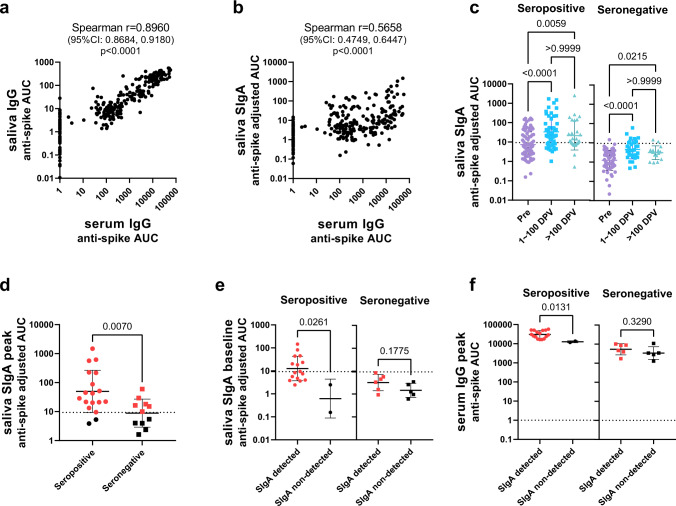
Mucosal SIgA antibody response to vaccination in adult participants with or without previous SARS-CoV-2 infection. **a**, **b** Correlation between anti-spike serum IgG antibody titers and saliva IgG antibody titers (**a**) or mucosal SIgA antibody titers (**b**) of samples collected from both seropositive and seronegative individuals at all time points. A total of 264 data points (6–13 data points per individual) were plotted for each panel. *X* and *Y*-axis values represent antibody titers (AUC: area under the curve). Spearman correlation coefficient and *p* values (two-tailed) are labeled above graphs. **c** Comparison of anti-SARS-CoV-2 spike SIgA titers in saliva between samples collected during different timepoints: before first vaccination (Pre, 91 samples in seropositive group and 62 samples in seronegative group), 1–100 days post first vaccination (1–100 DPV, 50 samples in seropositive group and 32 samples in seronegative group), and over 100 days post first vaccination (>100 DPV, 28 samples in seropositive group and 16 samples in seronegative group). Antibody titers (adjusted AUC) are plotted for each group. (statistical test: one-way ANOVA with Kruskal–Wallis test, *p* values above brackets). **d** Comparison of peak anti-SARS-CoV-2 spike mucosal SIgA titers between seropositive and seronegative individuals. The highest anti-spike SIgA titer among all timepoints (the peak saliva SIgA adjusted AUC) was plotted per individual (18 in the seropositive group and 11 in the seronegative group). Red dots (16 in the seropositive group and 6 in the seronegative group) indicate individuals with peak SIgA titers above the cut-off value (SIgA detected). **e** Comparison of baseline pre-vaccination anti-SARS-CoV-2 spikes SIgA titers in saliva between SIgA detected (16 in seropositive group and 6 in seronegative group) and SIgA non-detected (2 in seropositive group and 5 in seronegative group) seropositive and seronegative individuals. **f** Comparison of peak anti-SARS-CoV-2 spike IgG titers in serum of SIgA detected or SIgA non-detected seropositive and seronegative individuals. Mann–Whitney test was used in (**d**–**f**) (*p*-value (two-tailed) above bracket). All scatter plots in this figure are presented with geometric mean with geometric SD and a dotted line indicating the cut-off value (mean+3SD of the AUC of all samples collected from pre-vaccinated seronegative individuals). Source data are provided as a Source Data file.

Next, we performed correlation analyses including all the samples tested to reveal the relationship between the different antibody titers. As expected, a positive correlation was observed between serum and saliva anti-spike IgG titers (Fig. 2a), reflecting the fact that the IgG antibodies measured in saliva were originally serum IgG antibodies that seeped into the mucosa from circulation. In contrast, titers of saliva anti-spike SIgA, which are not derived from the circulation but were produced at the mucosa, did not strongly correlate with serum IgG titers (Fig. 2b). Next, to evaluate the difference in antibody titers over time, we grouped the data based on the collection timepoints: before first vaccination, 1–100 days post first vaccination, and more than 100 days post first vaccination. As anticipated serum IgG and mucosal SIgA antibody titers were significantly higher in seropositive individuals than in seronegative individuals before the first vaccination (Supplementary Fig. 3a–d). The serum and mucosal antibody response to the SARS-CoV-2 spike following vaccination remained significantly higher in seropositive individuals than in seronegative individuals (Supplementary Fig. 3a–c). In contrast, the mucosal anti-NP SIgA antibody response was significantly higher in samples collected from previously infected individuals than those from previously naive individuals only before vaccination and 1–100 days post first vaccination. This difference was not observed in samples collected after 100 days post first vaccination (Supplementary Fig. 3d). This indicated that the anti-NP SIgA response observed in seropositive individuals was not boosted by vaccination, and waned over time. Comparison between the different timepoints revealed that serum and mucosal anti-spike antibody titers significantly increased after vaccination in both seropositive and seronegative individuals (Fig. 2c and Supplementary Fig. 3e, f). In contrast, an increase in mucosal anti-NP IgA titers over time was not observed in either group (Supplementary Fig. 3g).

### SIgA is efficiently induced in seropositive individuals with higher pre-exisiting SIgA levels

In order to identify study participants who were able to successfully induce SIgA responses by vaccination, peak mucosal SIgA titers (the highest SIgA titer observed after vaccination) were assessed. Peak saliva SIgA titers were significantly higher in the seropositive individuals than the seronegative individuals (Fig. 2d). The same was observed for serum IgG titers (Supplementary Fig. 4a), which was consistent with a previous report^13^. 16/18 seropositive individuals presented peak SIgA titers above the cut-off value (mean+3 SD of the AUC for all samples collected from pre-vaccinated seronegative individuals), whereas only 6/11 seronegative individuals did (Fig. 2d). To identify whether pre-vaccination antibody titers were associated with induction of SIgA by vaccination, baseline antibody titers (measured using samples collected on the nearest possible date before vaccination), were evaluated. In order to assess this, we separated the seropositive and seronegative groups each into two categories: SIgA detected (those with peak saliva SIgA titers above the cut-off value) and SIgA non-detected (those with peak saliva SIgA titers below the cut-off value) individuals (Fig. 2d). In the seropositive group, baseline anti-SARS-CoV-2 spike saliva SIgA titers were significantly higher in SIgA detected individuals than SIgA non-detected individuals before vaccination (Fig. 2e). Significant differences were not observed in baseline anti-SARS-CoV-2 spike serum IgG and anti-SARS-CoV-2 NP saliva SIgA titers between SIgA detected and non-detected individuals (Supplementary Fig. 4b, c). However, for seronegative individuals, no significant difference was observed in baseline saliva anti-SARS-CoV-2 spike SIgA titers (Fig. 2e). Next, in order to assess whether pre-existing antibodies affect the magnitude of the antibody response induced by vaccination, correlation analyses between fold-increases in antibody titers by vaccination and baseline antibody titers were conducted. As a result, baseline saliva SIgA and serum IgG levels did not correlate with fold-increase in saliva SIgA levels, whereas baseline serum IgG antibody titers negatively correlated with fold-increase in serum IgG levels by vaccination (Supplementary Fig. 4d–f).

It may be possible that mucosal immunity induced by seasonal coronaviruses (HCoVs) is boosted by vaccination and that this cross-reactive immunity (which mostly targets S2^14,15^, with cross-reactivity being common between betacoronaviruses OC43, HKU1, and SARS-CoV-2) is responsible for the weak induction of SIgA found in the previously seronegative individuals. To evaluate this, SIgA binding activity against the spike proteins of betacoronaviruses HCoV-OC43 and HCoV-HKU1, and the antigenically conserved SARS-CoV-2 S2 domain were assessed. However, no significant differences in baseline anti-HCoV-OC43, HCoV-HKU1, and SARS-CoV-2 S2 saliva SIgA titers between SIgA detected and non-detected groups were observed in seronegative individuals (Supplementary Fig. 5a). In addition, the S2/RBD ratio of peak saliva SIgA was not significantly different between SIgA-induced seropositive and seronegative individuals, suggesting no skewing towards cross-reactive S2 antibodies (Supplementary Fig. 5b). In addition, the fold-increase of anti-SARS-CoV-2 spike SIgA levels by vaccination was significantly higher than that of anti-HCoV-OC43 and HCoV-HKU1 spike SIgA titers in SIgA-induced seropositive and seronegative individuals (Supplementary Fig. 5c). These data indicate that the saliva SIgA antibodies induced by vaccination in seronegative individuals were not back-boosted antibodies against HCoVs, but rather SARS-CoV-2 spike-specific antibodies that were newly elicited by vaccination. To assess whether the level of systemic immune response induced was associated with successful SIgA induction, peak serum anti-SARS-CoV-2 spike IgG antibodies were assessed. As a result, SIgA detected individuals presented significantly higher peak serum anti-SARS-CoV-2 spike IgG titers than SIgA non-detected individuals in the seropositive group but not in the seronegative group (Fig. 2f). Interestingly, there were no significant difference between the peak serum anti-SARS-CoV-2 spike IgG titers observed in SIgA non-detected seropositive individuals and SIgA detected seronegative individuals (*p* = 0.0714, Mann–Whitney test). Of note, neither the type of vaccine administered, sex, nor age influenced the induction of anti-SARS-CoV-2 spike saliva SIgA antibodies upon immunization (Supplementary Fig. 6a–c).

## Discussion

Our results support observations from other groups suggesting that intramuscular mRNA vaccination can induce SIgA antibodies in saliva under certain circumstances^16,17^. Our data further suggest that vaccination alone can induce a weak mucosal SIgA response in individuals without pre-existing mucosal antibody response to SARS-CoV-2, although SIgA induction is more efficient in individuals with pre-existing mucosal antibody response to SARS-CoV-2 elicited by COVID-19. Seropositive individuals presented significantly higher pre-vaccination baseline anti-SARS-CoV-2 spike saliva SIgA titers in SIgA detected individuals than SIgA non-detected individuals, whereas pre-vaccination baseline anti-SARS-CoV-2 spike saliva SIgA titers were not significantly different between SIgA detected and SIgA non-detected seronegative individuals. These observations suggested that vaccinated previously infected individuals were able to induce a relatively quick and high anti-spike SIgA response by boosting the pre-existing mucosal anti-SARS-CoV-2 spike immunity. In contrast, seronegative individuals did not have pre-existing anti-SARS-CoV-2 or cross-reacting anti-HCoV SIgA antibodies prior to vaccination, and thus weak anti-SARS-CoV-2 SIgA antibodies were elicited by vaccination in only approximately half of the individuals.

The mechanism by which intramuscularly administered vaccines induced a mucosal SIgA response remains unclear. There are hypotheses suggesting that antigen-presenting cells activated at the peripheral lymph nodes could migrate to the MALT and elicit a mucosal antibody response, or that antigen-specific B cells that have proliferated at the peripheral lymph nodes could migrate to the mucosal sites under the influence of their homing receptors, where these cells would differentiate into plasma cells and secrete antibodies^18^. Alternatively, it has been reported that SARS-CoV-2 vaccine antigens have been detected in the plasma of the Moderna mRNA-1273 vaccinees^19^, suggesting that vaccine antigens may have reached the MALT to elicit mucosal immune responses. While we did not detect a correlation between seasonal betacoronavirus immunity and induction of SIgA in naïve vaccinated individuals, it could be that our methods were not sensitive enough and/or our sample size was too small to discover a relationship. Thus, there is still a possibility that the SIgA responses observed here were a recall of immune memory elicited by past HCoV infections, which may cross-react with SARS-CoV-2 spike.

Peak serum anti-SARS-CoV-2 spike IgG titers were significantly higher in SIgA detected individuals than SIgA non-detected individuals in the previously infected group, indicating that the degree of immunological impact of vaccination on systemic immunity influences the induction efficiency of SIgA at the mucosa. However, there were no significant differences in peak serum anti-SARS-CoV-2 spike IgG titers between SIgA non-detected seropositive individuals and SIgA detected seronegative individuals, and between SIgA detected and non-detected individuals in the seronegative group. This suggested that SIgA induction cannot be explained solely by a strong immunological impact on systemic immunity, but other mechanisms (boosting of pre-existing mucosal immunity for seropositive individuals, and unknown mechanisms for seronegative individuals) are responsible for SIgA induction by vaccination.

It has been reported in past studies that preexisting serum antibodies induced by past vaccinations or infections can affect the magnitude of the antibody response induced by subsequent vaccinations, due to the masking of antigenic sites on vaccine antigens by pre-existing antibodies^20–22^. Indeed, a clear negative correlation could be observed between baseline serum IgG levels and fold-increase in serum IgG levels by vaccination in the current study. In contrast, baseline saliva SIgA levels did not correlate -positively or negatively - with a fold-increase in saliva SIgA levels. This can be explained by the fact that since SIgA is present in the mucosa, pre-existing SIgA will not be able to interact with vaccine antigens and affect the magnitude of SIgA response induced by vaccination. Since the mechanism underlying SIgA induction by vaccination we see in the current study is possibly activation of memory B cells upon re-exposure to cross-reactive antigen, it is expected that pre-existing serum IgG will affect the magnitude of saliva SIgA response as well. However, baseline serum IgG levels did not correlate with a fold-increase in saliva SIgA as well. This may be due to a lack of sensitivity in the SIgA detection assay, or a lack of data points, but further studies are needed in order to confirm this point.

Of note, among participants in the longitudinal observational Protection Associated with Rapid Immunity to SARS-CoV-2 (PARIS) study, breakthrough infection cases after vaccination have - in the pre-Omicron era - only been identified in individuals without SARS-CoV-2 infection prior to vaccination, and not in individuals with SARS-CoV-2 infection prior to vaccination (personal communication, data not yet published). It has also been reported that vaccinated individuals who experience breakthrough infections have significantly lower serum IgA levels compared to those who do not^16^. These, together with observations from the current study, may indicate that previously infected individuals mount mucosal SIgA antibody levels sufficient to provide protection from disease in response to vaccination, at least against antigenically matched variants. However, further studies are needed in order to confirm this and to determine the level of SIgA needed in order to prevent infection or transmission. SIgA induction in individuals is expected to be beneficial in various aspects. First, it has been demonstrated that dimerization of IgA antibodies presented higher virus neutralization activity compared to monomeric IgA of anti-SARS-CoV-2 antibodies^23^. Second, IgA multimerization could increase the cross-reactivity of antibodies, which may allow SIgA antibodies to provide better protection against antigenic variant viruses^4,24^. Thus, vaccination strategies, such as intranasal vaccines like NDV-HXP-S, that could successfully induce SIgA, should be sought for the control of the SARS-CoV-2 pandemic^25–27^. Further studies are needed to reveal the detailed mechanism of mucosal antibody induction by mRNA vaccination, determine SIgA titers that would provide sterilizing immunity, and evaluate the SIgA antiviral function in comparison to monomeric IgA.

## Methods

### Human samples (participant enrollment, serum and saliva collection, and methods)

Serum and saliva samples were collected from study participants in the longitudinal observational Protection Associated with Rapid Immunity to SARS-CoV-2 (PARIS) study. The study was reviewed and approved by the Mount Sinai Hospital Institutional Review Board (IRB-20-03374). All participants signed written consent forms prior to sample and data collection. All participants provided permission for sample banking and sharing. Saliva was collected in a cup and serum by venipuncture. The participants did not receive compensation. All biospecimen were coded and stored at −80 °C.

### Recombinant proteins

Recombinant SARS-CoV-2 proteins were produced using a mammalian cell protein expression system. SARS-CoV-2 spike (S), receptor binding domain (RBD), and nucleoprotein (NP) gene sequences (GenBank: MN908947 [https://www.ncbi.nlm.nih.gov/nuccore/MN908947]) were cloned into a mammalian expression vector, pCAGGs. The expression plasmids encoding for the spike of common human coronavirus (HCoV) OC43 and HKU1 were obtained from the NIH. S and RBD were produced as described previously^28,29^. Proteins were expressed using the Expi293 Expression System (Thermo Fisher Scientific), according to the manufacturer’s instructions. For S and RBD proteins, cell culture supernatants were collected and clarified by centrifugation at 4000 × *g*, filtered, and purified with Ni^2+^-nitrilotriacetic acid (NTA) agarose (QIAGEN). For NP, cell pellets were collected, and resuspended in high salt buffer (1 M Tris–hydrochloric acid (HCl), pH 8.0; 0.5 M ethylenediaminetetraacetic (EDTA) pH 8.0; 5 M NaCl; Triton X-100 and distilled water) and lysed using a sonicator. Lysed cells were then centrifuged and the supernatant was purified with Ni-NTA agarose (QIAGEN). The purified proteins were concentrated using Amicon Ultracell (Merck) centrifugation units, and the buffer was changed to phosphate-buffered saline (PBS, pH 7.4). Proteins were stored at −80 °C until use. SARS-CoV-2 S2 protein was purchased from Sino Biological (#40590-V08B).

### Antigen-specific serum IgG ELISA

Serum IgG ELISAs against SARS-CoV-2 spike were conducted as described previously^28,29^. Immulon 4 HBX 96-well microtiter plates (Thermo Fisher Scientific) were coated overnight at 4 °C with recombinant protein (100 ng/well) in PBS (pH 7.4). Well contents were discarded and wells blocked with 200 µL of 3% non-fat milk (AmericanBio) in PBS containing 0.1% Tween-20 (PBST) for one hour at room temperature (RT). After blocking, 100 µL of serum samples diluted (starting at 1:80 and serially diluted three-fold) with 1% non-fat milk in PBST was added to each well for reaction at RT for 2 h. After washing with PBST three times, 50 μL of horseradish peroxidase (HRP)-labeled goat anti-human IgG antibody (Sigma-Aldrich, #A0170) diluted 3000-fold with 1% non-fat milk in PBST was added to each well and incubated at RT for 1 h. After washing with PBST three times, 100 μL of SIGMAFAST *o*-phenylenediamine dihydrochloride substrate solution (Sigma-Aldrich) was added to each well for reaction at RT for 10 min. The reaction was stopped by the addition of 50 μL of 3 M HCl. Optical density (OD) at 490 nm was measured using a Synergy 4 (BioTek) plate reader. Eight wells on each plate received no primary antibody (blank wells) and the optical density in those wells was used to assess the background (OD values of sample wells were adjusted by deducting the average of blank values plus three times the standard deviation of the blank values). Binding curves of each sample were generated by plotting the OD values for each sample concentration. Area under the curve (AUC) values were calculated from these binding curves for each sample. The assay was done once per sample due to the limited sample amount.

### Antigen-specific saliva SIgA and IgG ELISA

Immulon 4 HBX 96-well microtiter plates (Thermo Fisher Scientific) were coated overnight at 4 °C with recombinant protein (100 ng/well) in PBS (pH 7.4). Well contents were discarded and wells blocked with 200 µL of 5% non-fat milk (AmericanBio) in PBST for one hour at RT. After blocking, 50 µL of saliva samples diluted (starting at 1:2 and serially diluted two-fold) with 2.5% non-fat milk in PBST was added to each well. For IgG measurement, the reaction of samples with antigen was conducted at RT for 2 h. After washing with PBST three times, 50 μL of HRP-labeled goat anti-human IgG (H + L) antibody (Fisher Scientific, #PI31412) diluted to 0.32 μg/mL with 2.5% non-fat milk in PBST was added to each well, and incubated at RT for 1 h. For SIgA measurement, the reaction of samples with antigen was conducted at 4 °C overnight. After washing with PBST three times, 50 μL of mouse anti-human secretory IgA antibody (MilliporeSigma, #HP6141) diluted to 5 μg/mL with 2.5% non-fat milk in PBST was added to each well, and incubated at RT for 2 h. These plates were washed again with PBST three times, and 50 μL of HRP-labeled goat anti-mouse IgG Fc antibody (Invitrogen, #31439) diluted 1:1000 with 2.5% non-fat milk in PBST was added to each well, and incubated at RT for 1 h. After washing with PBST three times, 100 μL of SIGMAFAST *o*-phenylenediamine dihydrochloride substrate solution (Sigma-Aldrich) was added to each well for reaction at RT for 10 min. The reaction was stopped by the addition of 50 μL of 3 M HCl. Optical density at 490 nm was measured using a Synergy 4 (BioTek) plate reader. Eight wells on each plate received no primary antibody (blank wells) and the optical density in those wells was used to assess the background (OD values of sample wells were adjusted by deducting the average of blank values plus three times the standard deviation of the blank values). Binding curves of each sample were generated by plotting the OD values for each sample concentration. Area under the curve (AUC) values were calculated from these binding curves for each sample. Antigen-specific SIgA AUC values were adjusted by dividing the values by total IgA concentration within saliva samples (adjusted AUC). The assay was done once per sample due to the limited sample amount.

### Measurement of total IgA concentration within saliva samples

Immulon 4 HBX 96-well microtiter plates (Thermo Fisher Scientific) were coated overnight at 4 °C with 250 ng/well of goat anti-human IgA (Bethyl Laboratories, #A80-102A) in PBS (pH 7.4). Well contents were discarded and blocked with 200 µL of 5% non-fat milk (AmericanBio) in PBST for one hour at RT. After blocking, 50 µL of serum samples diluted (starting at 1:256 and serially diluted three-fold) with 2.5% non-fat milk in PBST was added to each well and incubated at RT for 2 h. After washing with PBST three times, 50 μL of HRP-labeled goat anti-human IgA antibody (Bethyl Laboratories, #A80-102P) diluted to 1:10,000 with 2.5% non-fat milk in PBST was added to each well, and incubated at RT for 1 h. After washing with PBST three times, 100 μL of SIGMAFAST *o*-phenylenediamine dihydrochloride substrate solution (Sigma-Aldrich) was added to each well for reaction at RT for 10 min. The reaction was stopped by the addition of 50 μL of 3 M hydrochloric acid (HCl). Optical density at 490 nm was measured using a Synergy 4 (BioTek) plate reader. Purified human plasma IgA (EMD Millipore, #401098) was diluted (starting at 500 ng/mL and serially diluted two-fold) and was used as standard. A five-parameter logistic fit was conducted on the standard curve and IgA concentrations within saliva samples were calculated. The assay was done once per sample due to the limited sample amount.

### Statistical analysis

Differences between antibody titers between two groups were analyzed with the Mann–Whitney test. Differences between antibody titers between three groups were analyzed with one-way ANOVA with a Kruskal–Wallis test. Correlations between antibody titers were analyzed using Spearman’s rank test. All statistical analyses, AUC calculation, and five-parameter logistic fit of standard curves were conducted with Graphpad Prism version 9.

### Reporting summary

Further information on research design is available in the Nature Research Reporting Summary linked to this article.

## Supplementary information

Supplementary Information

Reporting Summary

## Data Availability

All data described here are provided in the source data accompanying this paper. Source data are provided with this paper.

## Source data

Source Data
