# Supplementary material for: SARS-CoV-2 vaccination induces mucosal antibody responses in previously infected individuals
Source: Nat Commun. 2022 Sep 1;13:5135. doi: 10.1038/s41467-022-32389-8 (PMC9435409; doi:10.1038/s41467-022-32389-8)
Supplement: Supplementary file 1 — Supplementary Information [file 41467_2022_32389_MOESM1_ESM.pdf]

Supplementary Information

Efficient mucosal antibody response to SARS-CoV-2 vaccination is induced in previously infected individuals

Supplementary Figures 1-6

Supplementary Tables 1-3

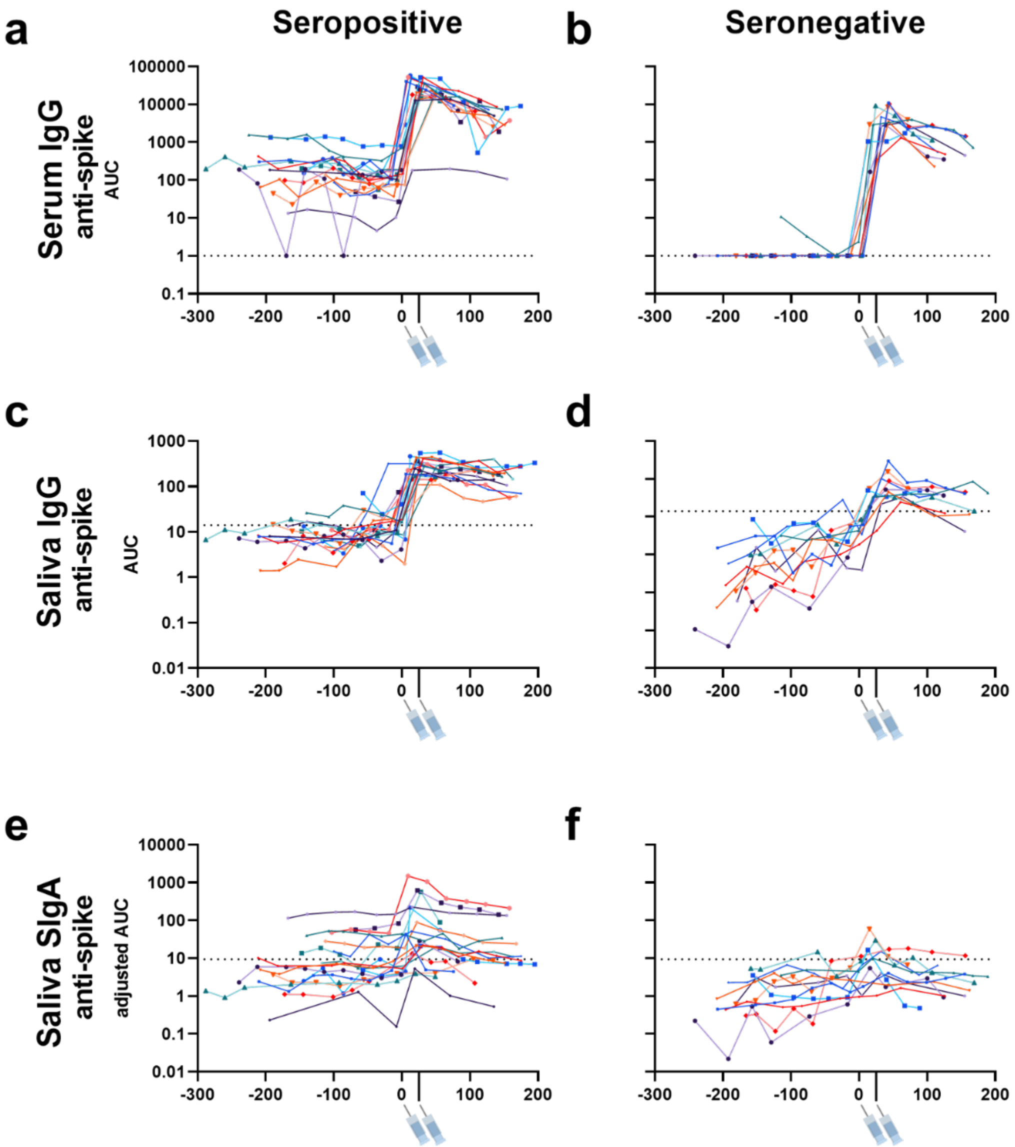

Supplementary Figure 1. Time course of vaccine-induced serum and mucosal anti-SARS-CoV-2 antibody titers in adult participants with or without previous SARS-CoV-2 infection.

Anti-SARS-CoV-2 spike IgG titers in serum (a, b), anti-SARS-CoV-2 spike IgG titers in saliva (c, d), and anti-SARS-CoV-2 spike SIgA titers in saliva (e, f) collected from 18 individuals with previous SARS-CoV-2 infection (seropositive) and 11 individuals without previous SARS-CoV-2 infection (seronegative) prior to and after vaccination. 6~13 different timepoints were assessed per individual. X-axis values represent days post first vaccination and Y-axis values represent antibody titers calculated as AUC (area under the curve). Syringe symbols point to the approximate time when the first and second vaccine dose were administered (28 days for Moderna mRNA-1273 vaccine and 21 days for the Pfizer-BioNTech BNT162b2 vaccine). Dotted lines are cut off values (mean+3SD of the AUC of samples from pre-vaccinated seronegative individuals). Different colors represent different individuals. Source data are provided as a Source Data file.

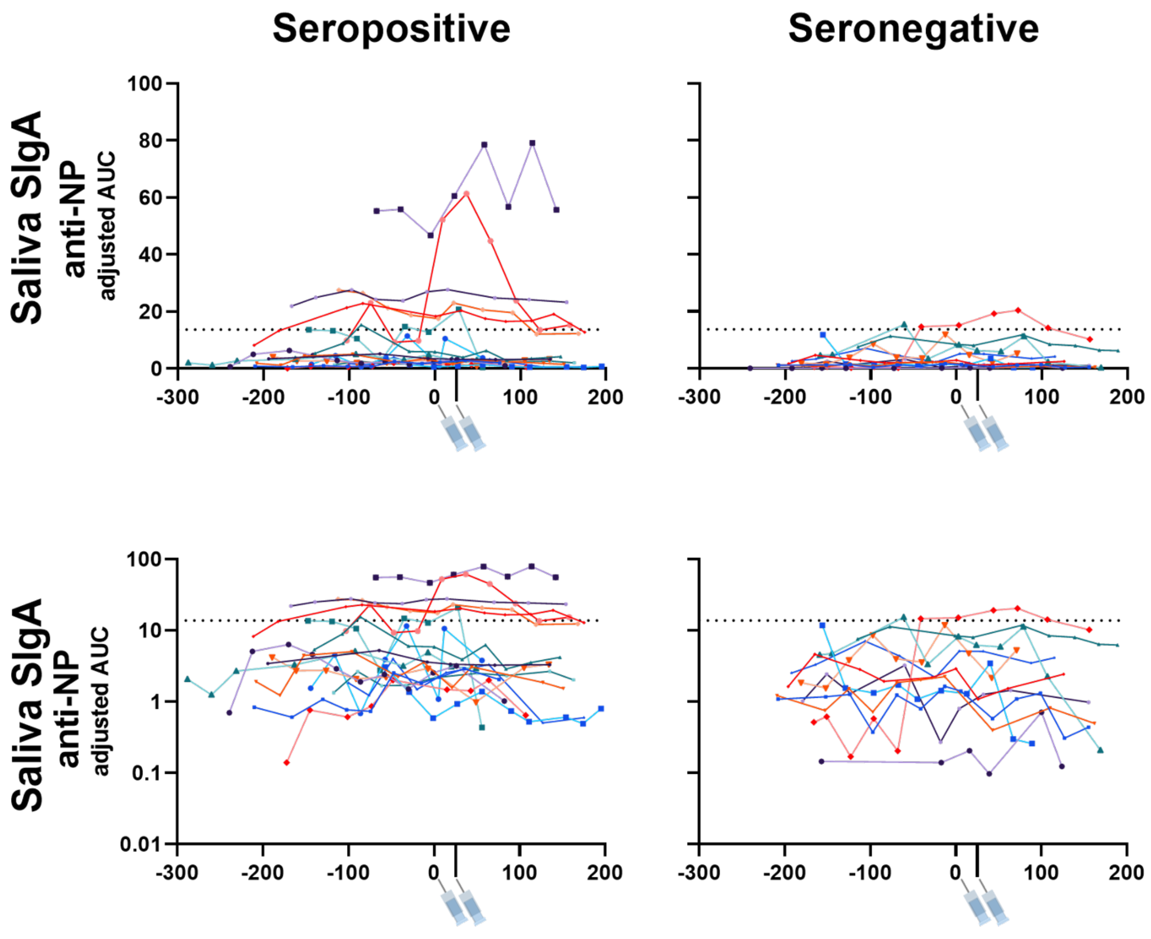

Supplementary Figure 2. Mucosal antibody response to SARS-CoV-2 NP in humans with or without previous SARS-CoV-2 infection.

Anti-SARS-CoV-2 NP SIgA titers in saliva collected from 18 individuals with previous SARS-CoV-2 infection (seropositive) and 11 individuals without previous SARS-CoV-2 infection (seronegative) prior to and after vaccination. 6~13 different timepoints were assessed per individual. X-axis values are days post first vaccination and Y-axis values are antibody titers calculated as AUC (area under the curve). Data is presented in linear and logarithmic scale in top and bottom panels, respectively. Syringe symbols point to the approximate time when the first and second vaccine dose was administered (28 days for Moderna mRNA-1273 vaccine and 21 days for the Pfizer-BioNTech BNT162b2 vaccine). The dotted line indicates the cut-off value (mean+3SD of the AUC of samples from pre-vaccinated seronegative individuals). Source data are provided as a Source Data file.

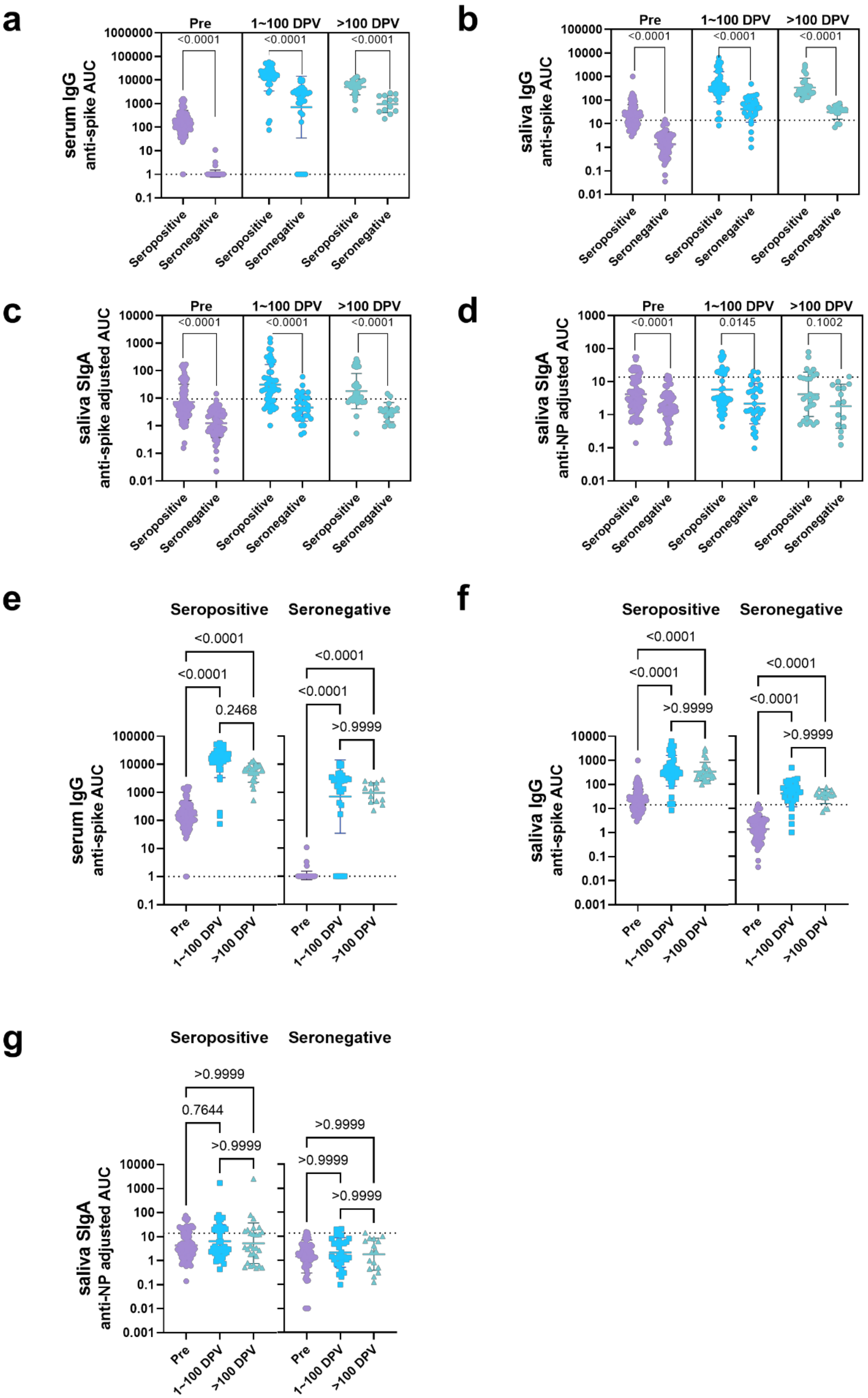
a
serum IgG anti-spike AUC
Pre
1~100 DPV
>100 DPV
<0.0001
<0.0001
<0.0001
1000000
100000
10000
1000
100
10
1
0.1
Seropositive
Seronegative
b
saliva IgG anti-spike AUC
Pre
1~100 DPV
>100 DPV
<0.0001
<0.0001
<0.0001
100000
10000
1000
100
10
1
0.1
0.01
Seropositive
Seronegative
c
saliva SIgA anti-spike adjusted AUC
Pre
1~100 DPV
>100 DPV
<0.0001
<0.0001
<0.0001
10000
1000
100
10
1
0.1
0.01
Seropositive
Seronegative
d
saliva SIgA anti-NP adjusted AUC
Pre
1~100 DPV
>100 DPV
<0.0001
0.0145
0.1002
1000
100
10
1
0.1
0.01
Seropositive
Seronegative
e
Seropositive
Seronegative
serum IgG anti-spike AUC
<0.0001
<0.0001
0.2468
<0.0001
<0.0001
>0.9999
100000
10000
1000
100
10
1
0.1
Pre
1~100 DPV
>100 DPV
f
Seropositive
Seronegative
saliva IgG anti-spike AUC
<0.0001
<0.0001
>0.9999
<0.0001
<0.0001
>0.9999
10000
1000
100
10
1
0.1
0.01
0.001
Pre
1~100 DPV
>100 DPV
g
Seropositive
Seronegative
saliva SIgA anti-NP adjusted AUC
>0.9999
0.7644
>0.9999
>0.9999
>0.9999
>0.9999
10000
1000
100
10
1
0.1
0.01
0.001
Pre
1~100 DPV
>100 DPV

Supplementary Figure 3. Comparison of serum and mucosal antibody titers between different timepoints.

Comparison of anti-SARS-CoV-2 spike IgG titers in serum (a), anti-SARS-CoV-2 spike IgG titers in saliva (b), anti-SARS-CoV-2 spike SIgA titers in saliva (c), and anti-SARS-CoV-2 NP SIgA titers in saliva (d) between samples collected from seropositive and seronegative individuals. Samples were separated into three groups: those collected prior to first vaccination (Pre, purple), at 1~100 days post first vaccination (1~100 DPV, blue), and after 100 days post first vaccination (>100 DPV, green) (Mann-Whitney test, p value (two-tailed) above bracket). Comparison of anti-SARS-CoV-2 spike IgG titers in serum (e), anti-SARS-CoV-2 spike IgG titers in saliva (f), and anti-SARS-CoV-2 NP SIgA titers in saliva (g) between samples collected during the three different timepoints. Dotted lines are cut off values (mean+3SD of the AUC of samples from pre-vaccinated seronegative individuals). For the seropositive group, 170 serum samples (101 in Pre, 47 in 1~100 DPV, and 22 in >100 DPV) and 166 saliva samples (90 in Pre, 49 in 1~100 DPV, and 27 in >100 DPV) collected from 18 individuals were included in the analysis. For the seronegative group, 108 serum samples (63 in Pre, 32 in 1~100 DPV, and 13 in >100 DPV) and 110 saliva samples (62 in Pre, 32 in 1~100 DPV, and 16 in >100 DPV) collected from 11 individuals were included in the analysis. Data is presented in scatter plots with geometric mean with geometric SD. (One-way ANOVA with Kruskal-Wallis test, p values above brackets). Y-axis values are antibody titers calculated as AUC (area under the curve). Source data are provided as a Source Data file.

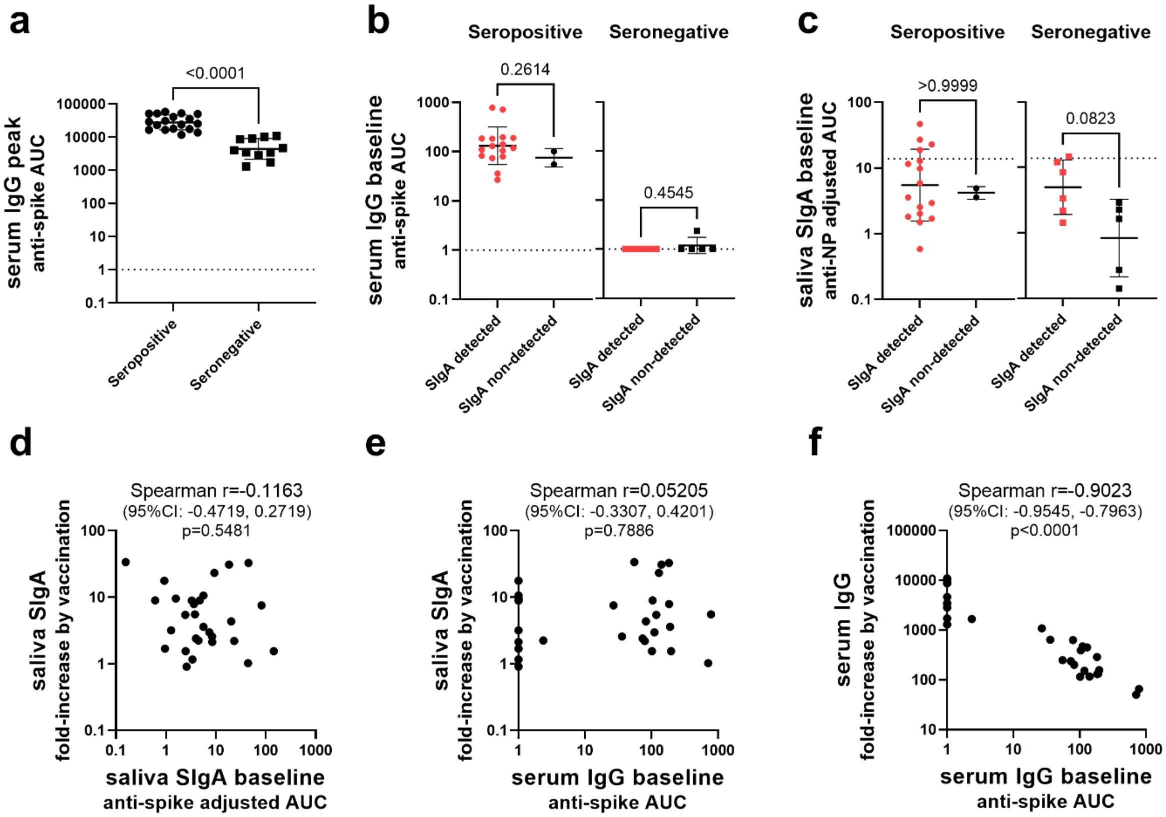

Supplementary Figure 4. Analyses of antibody response to vaccination in the context of pre-existing antibody titers

(a) Comparison of peak anti-SARS-CoV-2 spike serum IgG titers between 16 seropositive individuals and 11 seronegative individuals post vaccination (Mann-Whitney test, p value (two-tailed) above bracket). Data is presented in scatter plots with geometric mean with geometric SD. The dotted line indicates the cut-off value. (b, c) Comparison of anti-SARS-CoV-2 spike IgG titers in serum (b) and anti-NP SIgA titers in saliva (c) of samples collected on the nearest possible date before vaccination (baseline) between individuals who presented mucosal anti-spike SIgA antibody titers above the cut-off value SIgA detected (16 in seropositive group and 6 in seronegative group) and those who did not SIgA non-detected (2 in seropositive group and 6 in seronegative group) in seropositive and seronegative individuals (Mann-Whitney test, p values (two-tailed) above brackets). Data is presented in scatter plots with geometric mean with geometric SD. The dotted lines indicate the cut-off value (mean+3SD of the AUC of all samples collected from pre-vaccinated seronegative individuals for c). (d) Correlation analysis between baseline saliva SIgA titer and fold increase (peak antibody titer divided by baseline antibody titer) in saliva SIgA titer of the 18 seropositive and 11 seronegative individuals. Spearman correlation coefficient and p values (two-tailed) are labeled above graph. (e) Correlation analysis between baseline serum IgG titer and fold increase (peak antibody titer divided by baseline antibody titer) in saliva SIgA titer of the 18 seropositive and 11 seronegative individuals. Spearman correlation coefficient and p values (two-tailed) are labeled above graph. (f) Correlation analysis between baseline serum IgG titer and fold increase (peak antibody titer divided by baseline antibody titer) in serum IgG titer of the 18 seropositive and 11 seronegative individuals. Spearman correlation coefficient and p values (two-tailed) are labeled above graph. Source data are provided as a Source Data file.

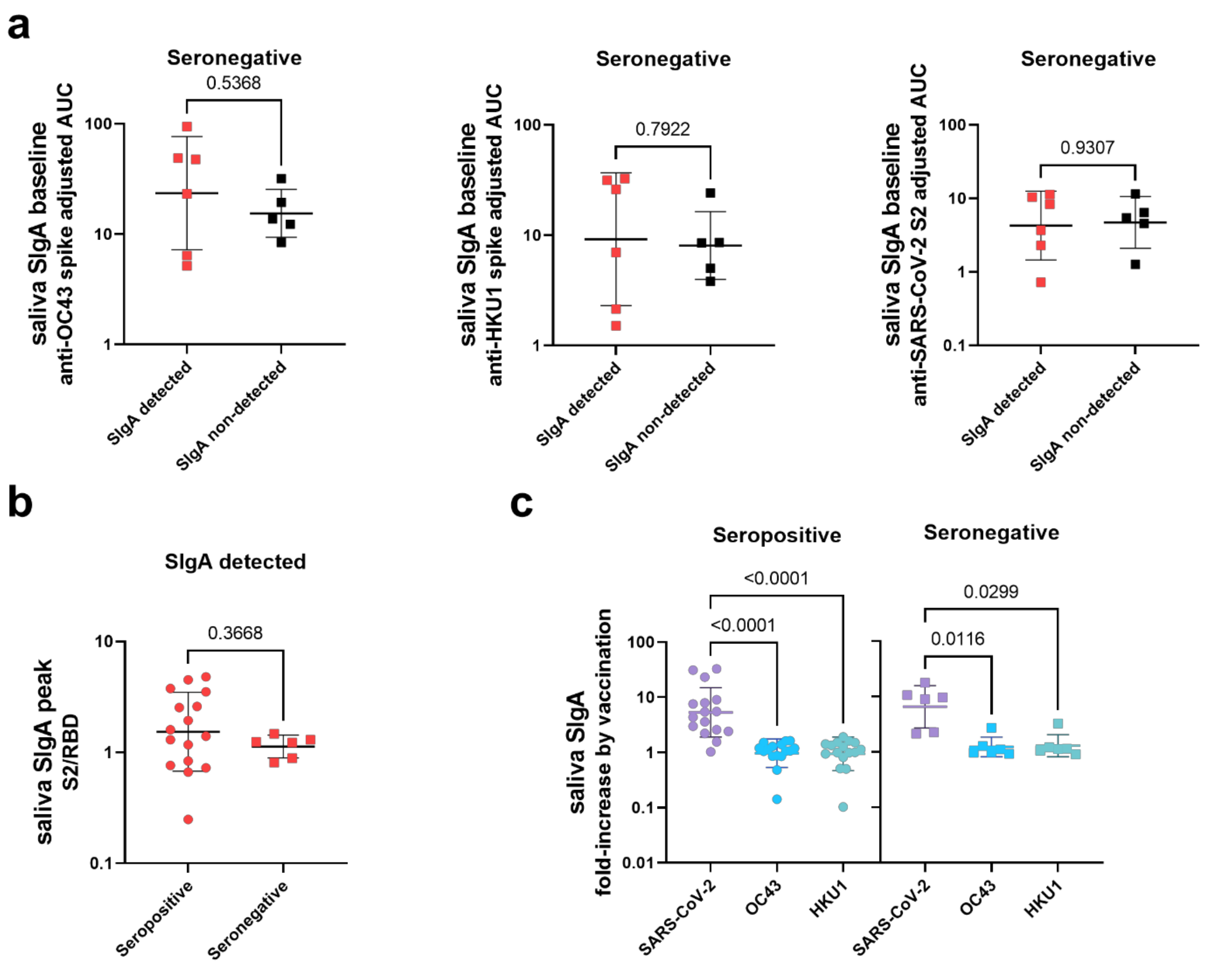

Supplementary Figure 5. Analyses of vaccine induced antibody binding to he spike proteins of betacoronaviruses HCoV-OC43 and HCoV-HKU1, and the antigenically conserved SARS-CoV-2 S2 domain

(a) Comparison of baseline anti-HCoV-OC43 spike (left panel), anti-HCoV-HKU1 spike (middle panel), and anti-SARS-CoV-2 spike S2 domain (right panel) reactivity between 6 SIgA induced and 5 SIgA non-induced seronegative individuals (Mann-Whitney test, p values (two-tailed) above brackets). (b) Comparison of peak saliva SIgA S2/RBD antibody ratio (calculated by dividing anti-SARS-CoV-2 S2 SIgA adjusted AUC by anti-SARS-CoV-2 RBD SIgA adjusted AUC) between 16 seropositive and 6 seronegative individuals who successfully induced SIgA in response to vaccination (Mann-Whitney test, p value (two-tailed) above bracket). (c) Comparison of fold increase in anti-SARS-CoV-2, anti-HCoV-OC43, and anti-HCoV-HKU1 SIgA titers in saliva by vaccination in 16 seropositive and 6 seronegative individuals who successfully induced SIgA in response to vaccination (One-way ANOVA with Kruskal-Wallis test, p values above brackets). Fold increase was calculated by dividing peak SIgA adjusted AUC by baseline SIgA adjusted AUC. All data is presented in scatter plots with geometric mean with geometric SD. Source data are provided as a Source Data file.

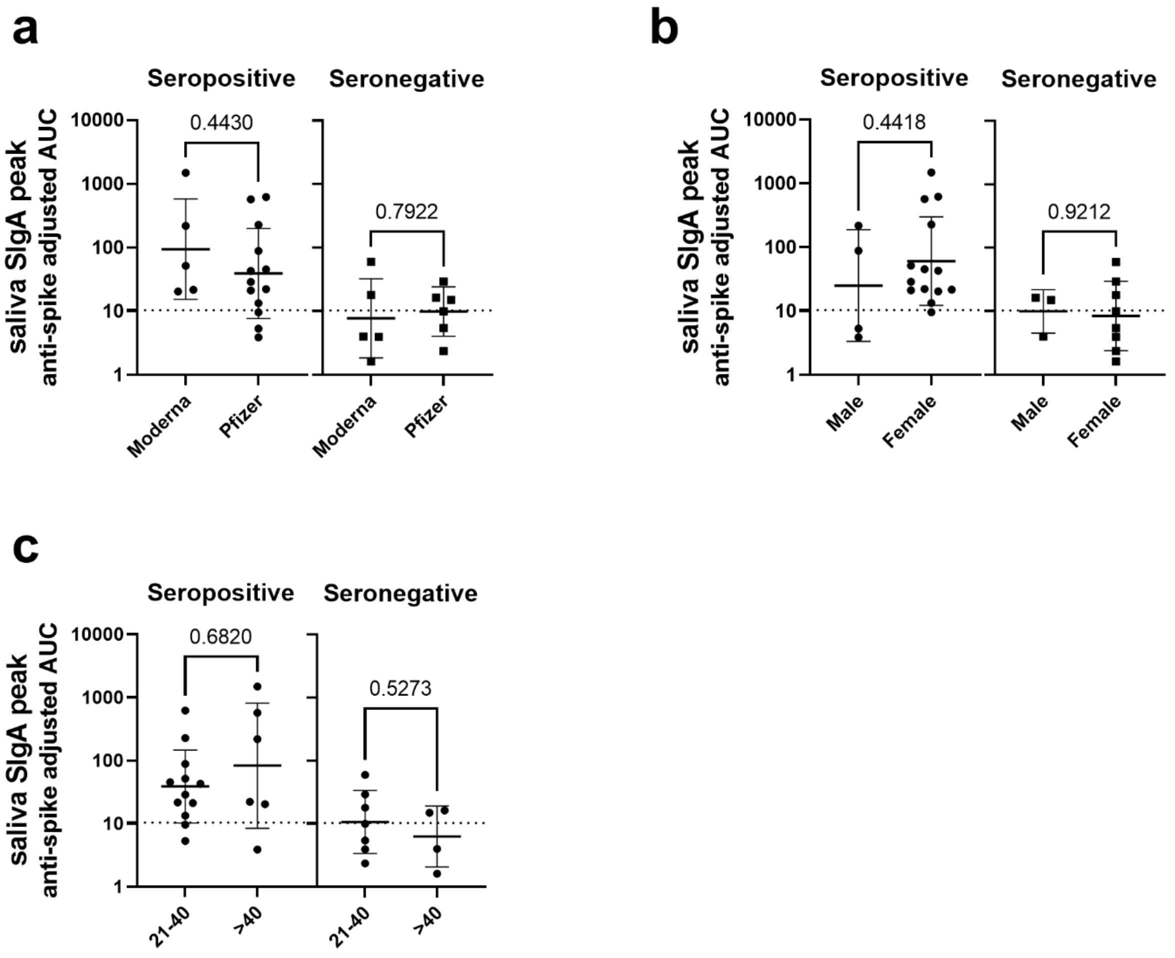

Supplementary Figure 6. Analyses of antibody response to vaccination in the context of demographics of study participants.

(a) Comparison of peak anti-SARS-CoV-2 spike SIgA titers between recipients of the Moderna mRNA-1273 vaccine (Moderna, 5 in seropositive group and 5 in seronegative group) and the Pfizer-BioNTech BNT162b2 vaccine (Pfizer, 13 in seropositive group and 6 in seronegative group) in seropositive and seronegative individuals (Mann-Whitney test, p values (two-tailed) above brackets). (b) Comparison of peak anti-SARS-CoV-2 spike SIgA titers between male (4 in seropositive group and 3 in seronegative group) and female (14 in seropositive group and 8 in seronegative group) individuals (Mann-Whitney test, p values (two-tailed) above brackets). (c) Comparison of peak anti-SARS-CoV-2 spike SIgA titers between age (Mann-Whitney test, p values (two-tailed) above brackets). Individuals were largely sorted into two age groups: 21~40 (12 in seropositive group and 7 in seronegative group) and >40 (6 in seropositive group and 4 in seronegative group). The dotted line indicates the cut-off value (mean+3SD of the AUC of all samples collected from pre-vaccinated seronegative individuals). All data is presented in scatter plots with geometric mean with geometric SD. Source data are provided as a Source Data file.

Supplementary Table 1. Comparison of statistical difference between seropositive and seronegative groups

	Seropositive (N=18)	Seronegative (N=11)	p value (two-tailed)
Sex (Male/Female)	4/14	3/8	>0.9999
Vaccine (Pfizer/Moderna)	13/5	6/5	0.4320
Age (Average <Min, Max>)	34.9 <21, 46>	40.6 <26, 62>	0.209
Serum samples (n, Average DPV <Min, Max>)	170, -28.08 <-288, 174>	109, -30.24 <-241, 167>	0.7977
Saliva samples (n, Average DPV <Min, Max>)	166, -16.31 <-288, 195>	110, -24.15 <-241, 189>	0.4984

DPV: Days post first vaccine. Fisher's exact test for analyses of sex and vaccine type distribution. Mann-Whitney test for analyses of age and sample collection timepoint distribution. Source data are provided as a Source Data file.

Supplementary Table 2. Demographics of seropositive study participants and SIgA detection status

Participant number	Age	Sex	Vaccine type	SIgA detection after vaccination	Sample collection timepoints in day post first vaccine (*Serum collection only **Saliva collection only)													
1	36-40	Male	Pfizer	non-detected	-194	-64	-8	19	71	135								
2	31-35	Female	Pfizer	detected	-210	-166	-130	-102	-74	-47	-15	6	35	76				
4	31-35	Female	Pfizer	detected	-225 *	-168 *	-140	-107	-86	-30	0	33	60	84	113	147		
5	21-25	Female	Moderna	detected	-208	-180	-152	-96	-42	-12	21	44	72 *	127	151			
6	41-45	Female	Pfizer	detected	-211	-182	-103	-84	-50 *	-29 *	1	31	59	83	113	139	175 **	
9	31-35	Female	Pfizer	detected	-239	-212	-170	-142	-114	-86	-58	-30	-1	26	82			
11	31-35	Female	Pfizer	detected	-193 *	-141 *	-113 *	-87 *	-57	-29	-1	27	56	90	111	154	174	195 **
14	41-45	Male	Pfizer	non-detected	-288	-260	-231	-163	-92	-36	-7	20	49 **					
16	36-40	Female	Pfizer	detected	-189	-161	-126	-91	-56	-35 *	-7	49	105	134				
17	31-35	Female	Pfizer	detected	-172	-145	-101	-73	-48	-18 *	15	43	64	107				
21	21-25	Female	Pfizer	detected	-167	-139	-97	-69	-37	-9	15	70	110	154 **				
23	31-35	Female	Moderna	detected	-100	-76	-48	-20	14	64	127	175 **						
24	46-50	Female	Moderna	detected	-117	-89	-61	-33	2	51	85	135	163 **					
25	36-40	Male	Pfizer	detected	-112	-84	-55	-28	4	22	56	91	119	168 **				
26	46-50	Female	Moderna	detected	-103	-75	-47	-19	9	37	65	95	123	158				
27	26-30	Female	Pfizer	detected	-68	-40	-5	23	58	86	114	142						
28	46-50	Male	Moderna	detected	-144	-116	-86	-57	-32	5	12	56						
29	41-45	Female	Pfizer	detected	-147	-119	-91	-63	-35	-7	28	56						

Supplementary Table 3. Demographics of seronegative study participants and SIgA induction status

Participant number	Age	Sex	Vaccine type	SIgA detection after vaccination	Sample collection timepoints in day post first vaccine (*Serum collection only **Saliva collection only)												
3	56-60	Male	Moderna	non-detected	-208	-152	-124	-97	-69	-41	-13	6	43	71	99	127	155
7	26-30	Female	Pfizer	detected	-115	-77	-35 *	-1	20	77	109	139	167	189 **			
8	36-40	Female	Pfizer	non-detected	-209	-153	-125	-97	-69	-41	-13	43	110	162 **			
10	61-65	Female	Moderna	non-detected	-196	-165	-112	-84	-56 *	-28	0	26	61	126			
12	36-40	Female	Pfizer	non-detected	-241	-192	-157	-129	-73	-17	16	39	100	124			
13	46-50	Male	Pfizer	detected	-156	-129	-96	-67	-45	-17	13	40	67	89 **			
15	31-35	Female	Pfizer	detected	-159	-145	-61	-33	3	24	52	79	107	169 **			
18	31-35	Female	Moderna	detected	-181	-152	-125	-97	-70	-40	-13	15	42	71			
19	26-30	Female	Moderna	detected	-166	-151	-123	-96	-68	-41	3	44	72	107	156		
20	36-40	Female	Moderna	non-detected	-179	-151	-123	-60	-18	4	32	64	155				
22	61-65	Male	Pfizer	detected	-192	-164	-106	-66	-24	4	32	60 *	88	115			
