# Supplementary material for: SARS-CoV-2 vaccination induces mucosal antibody responses in previously infected individuals
Source: Nat Commun. 2022 Sep 1;13:5135. doi: 10.1038/s41467-022-32389-8 (PMC9435409; doi:10.1038/s41467-022-32389-8)
Supplement: Supplementary file 2 — Reporting Summary [file 41467_2022_32389_MOESM2_ESM.pdf]

nature research

Corresponding author(s): Florian Krammer

Last updated by author(s): May 30, 2022

Reporting Summary

Nature Research wishes to improve the reproducibility of the work that we publish. This form provides structure for consistency and transparency in reporting. For further information on Nature Research policies, see our Editorial Policies and the Editorial Policy Checklist.

Statistics

For all statistical analyses, confirm that the following items are present in the figure legend, table legend, main text, or Methods section.

n/a	Confirmed	
☐	☒	The exact sample size (n) for each experimental group/condition, given as a discrete number and unit of measurement
☐	☒	A statement on whether measurements were taken from distinct samples or whether the same sample was measured repeatedly
☐	☒	The statistical test(s) used AND whether they are one- or two-sided<br>*Only common tests should be described solely by name; describe more complex techniques in the Methods section.*
☒	☐	A description of all covariates tested
☒	☐	A description of any assumptions or corrections, such as tests of normality and adjustment for multiple comparisons
☐	☒	A full description of the statistical parameters including central tendency (e.g. means) or other basic estimates (e.g. regression coefficient) AND variation (e.g. standard deviation) or associated estimates of uncertainty (e.g. confidence intervals)
☐	☒	For null hypothesis testing, the test statistic (e.g. F, t, r) with confidence intervals, effect sizes, degrees of freedom and P value noted<br>*Give P values as exact values whenever suitable.*
☒	☐	For Bayesian analysis, information on the choice of priors and Markov chain Monte Carlo settings
☒	☐	For hierarchical and complex designs, identification of the appropriate level for tests and full reporting of outcomes
☐	☒	Estimates of effect sizes (e.g. Cohen's d, Pearson's r), indicating how they were calculated

Our web collection on statistics for biologists contains articles on many of the points above.

Software and code

Policy information about availability of computer code

Data collection	Agilent BioTeK Gen5 Microplate Reader and Imager Software (Version 3.09.07), Microsoft Excel (2021)
Data analysis	GraphPad Prism 9

For manuscripts utilizing custom algorithms or software that are central to the research but not yet described in published literature, software must be made available to editors and reviewers. We strongly encourage code deposition in a community repository (e.g. GitHub). See the Nature Research guidelines for submitting code & software for further information.

Data

Policy information about availability of data

All manuscripts must include a data availability statement. This statement should provide the following information, where applicable:

- Accession codes, unique identifiers, or web links for publicly available datasets
- A list of figures that have associated raw data
- A description of any restrictions on data availability

Source data are provided with this paper.

Field-specific reporting

Life sciences study design

All studies must disclose on these points even when the disclosure is negative.

Sample size	Sample size was determined/limited by available number of samples. The maximum number of samples available was analyzed.
Data exclusions	All data was included in the analysis.
Replication	Replication was not possible due to limited sample amount.
Randomization	We selected PARIS participants with and without documented SARS-CoV-2 infection based on the availability of longitudinal saliva samples (average 9.52 samples per participant). Since we studied SARS-CoV-2 antibodies over time in a given participant there was no need for randomization of study groups.
Blinding	Blinding was done at time of obtaining data, but not at time of data analysis, since the information was needed for data analyses.

Reporting for specific materials, systems and methods

We require information from authors about some types of materials, experimental systems and methods used in many studies. Here, indicate whether each material, system or method listed is relevant to your study. If you are not sure if a list item applies to your research, read the appropriate section before selecting a response.

Materials & experimental systems

n/a	Involved in the study	
☐	☒	Antibodies
☒	☐	Eukaryotic cell lines
☒	☐	Palaeontology and archaeology
☒	☐	Animals and other organisms
☐	☒	Human research participants
☒	☐	Clinical data
☒	☐	Dual use research of concern

Methods

n/a	Involved in the study	
☒	☐	ChIP-seq
☒	☐	Flow cytometry
☒	☐	MRI-based neuroimaging

Antibodies

Antibodies used

horseradish peroxidase (HRP)-labeled goat anti-human IgG antibody (Sigma-Aldrich, #A0170, Lot#97267), HRP-labeled goat anti-human IgG (H+L) antibody (Invitrogen, #PI31412, Lot#WG3244976), mouse anti-human secretory IgA antibody (MilliporeSigma, #411423, Lot#3700920, clone#HP6141), HRP labeled goat anti-mouse IgG Fc antibody (Invitrogen, #31439, Lot#WH3357711), HRP labeled goat anti-human IgA antibody (Bethyl Laboratories, #A80-102P, Lot#6), goat anti-human IgA (Bethyl Laboratories, #A80-102A, Lot#2)

Validation

horseradish peroxidase (HRP)-labeled goat anti-human IgG antibody: Specificity of the Anti-Human IgG (Fc specific)- Peroxidase is determined by ELISA. The conjugate is specific for human IgG (Fc fragment) when tested against human IgA, IgG (Fab and Fc fragments), IgM, Bence Jones kappa, and lambda myeloma proteins. Cross-reactivity of the antibody-conjugate is determined by ELISA. The conjugate shows no reactivity with mouse or rat IgG and and yields reduced background with mouse or rat samples.

HRP-labeled goat anti-human IgG (H+L) antibody: When tested by immunoelectrophoresis, this antibody reacts with the heavy chains of human IgG and with light chains common to most human immunoglobulins. No antibody was detected against non-immunoglobulin serum proteins. The antibody has been tested by ELISA and/or solid-phase adsorbed to ensure minimal cross-reaction with bovine, horse and mouse serum proteins. However, this antibody may cross-react with immunoglobulins from other species.

mouse anti-human secretory IgA antibody: This Mouse Anti-Human IgA, Secretory (HP6141) is validated for use in ELISA for the detection of Human IgA, Secretory. Cross-reactivity by ELISA against human myeloma proteins:
Human IgG: <0.01%
Human IgE: <0.01%
Human IgM: <0.01%
Human IgA1: <0.01%
Human IgA2: <0.01%
Human secretory IgA: 100%
Hamilton, R.G., 1990. Ann. Biol. Clin.48, 473.
Fasullo, F.J., et al. 1989. Clin. Chem.35, 364.
Reimer, C.B., et al. 1989. Immunol. Letters21, 209.

HRP labeled goat anti-mouse IgG Fc antibody: This antibody reacts with heavy chains on mouse IgG but not with the light chains on

most mouse immunoglobulins. No antibody was detected against non-immunoglobulin serum proteins or mouse IgM. The antibody has been tested by ELISA and/or solid-phase adsorbed to ensure minimal cross-reaction with human, bovine and horse serum proteins. However, this antibody may cross-react with immunoglobulins from other species.

HRP labeled goat anti-human IgA antibody: By immunoelectrophoresis and ELISA this antibody reacts specifically with human IgA. Cross reactivity with IgG, IgM and light chains is less than 0.1%. This antibody may cross react with IgA from other species.

goat anti-human IgA: By immunoelectrophoresis and ELISA this antibody reacts specifically with human IgA. Cross reactivity with IgG, IgM and light chains is less than 0.1%. This antibody may cross react with IgA from other species.

Human research participants

Policy information about studies involving human research participants

Population characteristics	Population characteristics are described in detail in Supplementary Table 2 and 3.
Recruitment	The PARIS cohort was started in April 2020. We enrolled and followed 501 health care workers. The study was open to any health care worker of the Mount Sinai Health System. The average age of the participants is 40 years as such the cohort may not reflect the general population. Given the longitudinal nature of the antibody analysis, each participant can serve as its own control.
Ethics oversight	Institutional Review Board of the Icahn School of Medicine at Mount Sinai

Note that full information on the approval of the study protocol must also be provided in the manuscript.
